# Microdeletion syndromes

**DOI:** 10.1186/1755-8166-7-S1-I13

**Published:** 2014-01-21

**Authors:** Chaitanya Datar

**Affiliations:** 1 Sahyadri Genetics, Unit of Sahyadri Hospitals, Barve Memorial Complex, J.M. Road, Pune, India

## 

Microdeletion syndromes are a group of disorders characterized by the deletion of a small chromosomal segment (usually <5 Mb in size) encompassing multiple disease genes, each potentially contributing to the disease phenotype independently. The mechanism of disease causation is usually due to haploinsufficiency of certain critical genes of that region. The genetic changes of these microdeletion syndromes are often not detected by the current band resolution using the routine or high resolution karyotyping (2-5 Mb) but require application of molecular cytogenetic techniques like Fluorescence *in-situ* Hybridization (FISH) or the latest array CGH technique.

FISH is now the standard technique for the diagnosis of common microdeletion syndromes like Prader Willi syndrome, Angelman syndrome, Velocardiofacial (DiGeorge) syndrome, William syndrome etc. It is also possible to diagnose rare syndromes like Wolf Hirschhorn syndrome, Smith Magenis syndrome etc by FISH if the degree of clinical suspicion is high. With the advent of chromosomal microarrays, detection of newer microdeletion syndromes and better characterization of existing syndromes has become possible.

